# Supplementary material for: Effect of Long-Term Adaptation to Cold and Short-Term Cooling on the Expression of the TRPM2 Ion Channel Gene in the Hypothalamus of Rats
Source: Curr Issues Mol Biol. 2023 Jan 20;45(2):1002–11. doi: 10.3390/cimb45020065 (PMC9955288; doi:10.3390/cimb45020065)
Supplement: Supplementary file 1 [file cimb-45-00065-s001.zip › cimb-2112570-supplementary.pdf]

current issues in molecular biology

Supplementary Materials

Effect of Long-Term Adaptation to Cold and Short-Term Cooling on the Expression of the TRPM2 Ion Channel Gene in the Hypothalamus of Rats

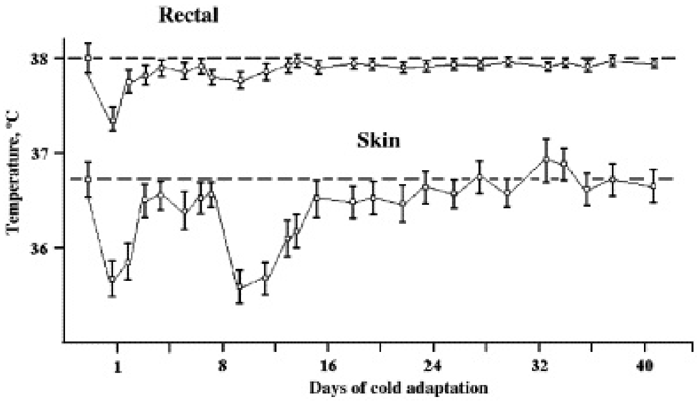

Figure S1. The time-courses of the skin temperature in the area of *n. sapheni* innervation and rectal temperature during 6 weeks adaptation to cold. Dashed lines represent the mean skin and rectal temperatures in control rats in the warm environment. The recordings of temperatures were daily made. Bars are SEM (from Kozyreva [38]).

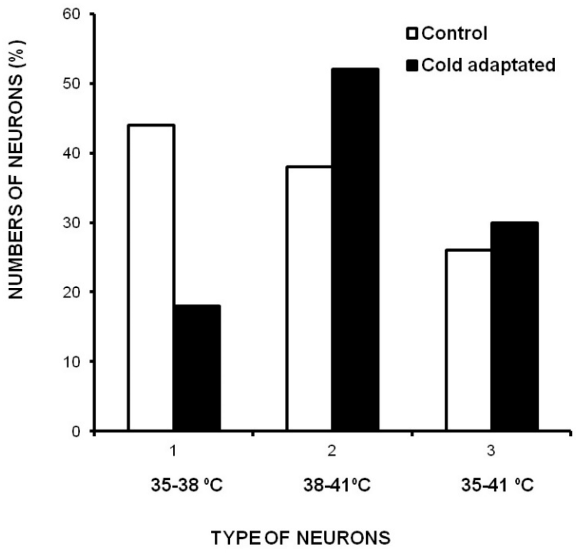

Figure S2. Number (%) of different types of neurons with different ranges of thermosensitivity (1, 2 and 3) in the medial preoptic area of hypothalamus in the control and cold-adapted rats. Diapasons of the neuron thermosensitivity are indicated at the bottom (from Kozyreva, Pierau [42], and Kozyreva [38] with modification).
